# Regulation of signaling genes by TGFβ during entry into dauer diapause in *C. elegans*

**DOI:** 10.1186/1471-213X-4-11

**Published:** 2004-09-20

**Authors:** Tao Liu, Karen K Zimmerman, Garth I Patterson

**Affiliations:** 1 Department of Molecular Biology and Biochemistry, Rutgers, the State University of New Jersey, Piscataway, NJ 08854, USA; 2 Cancer Institute of New Jersey, New Brunswick, NJ 08901, USA

## Abstract

**Background:**

When resources are scant, *C. elegans *larvae arrest as long-lived dauers under the control of insulin/IGF- and TGFβ-related signaling pathways. However, critical questions remain regarding the regulation of this developmental event. How do three dozen insulin-like proteins regulate one tyrosine kinase receptor to control complex events in dauer, metabolism and aging? How are signals from the TGFβ and insulin/IGF pathways integrated? What gene expression programs do these pathways regulate, and how do they control complex downstream events?

**Results:**

We have identified genes that show different levels of expression in a comparison of wild-type L2 or L3 larvae (non-dauer) to TGFβ mutants at similar developmental stages undergoing dauer formation. Many insulin/IGF pathway and other known dauer regulatory genes have changes in expression that suggest strong positive feedback by the TGFβ pathway. In addition, many insulin-like ligand and novel genes with similarity to the extracellular domain of insulin/IGF receptors have altered expression. We have identified a large group of regulated genes with putative binding sites for the FOXO transcription factor, DAF-16. Genes with DAF-16 sites upstream of the transcription start site tend to be upregulated, whereas genes with DAF-16 sites downstream of the coding region tend to be downregulated. Finally, we also see strong regulation of many novel hedgehog- and patched-related genes, hormone biosynthetic genes, cell cycle genes, and other regulatory genes.

**Conclusions:**

The feedback regulation of insulin/IGF pathway and other dauer genes that we observe would be predicted to amplify signals from the TGFβ pathway; this amplification may serve to ensure a decisive choice between "dauer" and "non-dauer", even if environmental cues are ambiguous. Up and down regulation of insulin-like ligands and novel genes with similarity to the extracellular domain of insulin/IGF receptors suggests opposing roles for several members of these large gene families. Unlike in adults, most genes with putative DAF-16 binding sites are upregulated during dauer entry, suggesting that DAF-16 has different activity in dauer versus adult metabolism and aging. However, our observation that the position of putative DAF-16 binding sites is correlated with the direction of regulation suggests a novel method of achieving gene-specific regulation from a single pathway. We see evidence of TGFβ-mediated regulation of several other classes of regulatory genes, and we discuss possible functions of these genes in dauer formation.

## Background

Changes in environmental conditions alter the physiology of all organisms. Evolution and experience create a signaling architecture that assesses current conditions and makes changes in physiology to attain the most appropriate state for a predicted future condition. The dauer larva of *C. elegans *forms when sensory inputs suggest that food resources will be inadequate for successful reproduction [1]. Food availability, competition for available food resources (population density, as measured by the concentration of a constitutively secreted pheromone), and temperature are assessed by identified chemosensory and thermosensory neurons, and signals are transduced from these neurons to affect the physiology and structure of most cell types in the body. Development is arrested after the second larval molt, and the third-stage larva that is formed is structurally and behaviorally specialized for dispersal and long-term survival.

**Figure 1:**
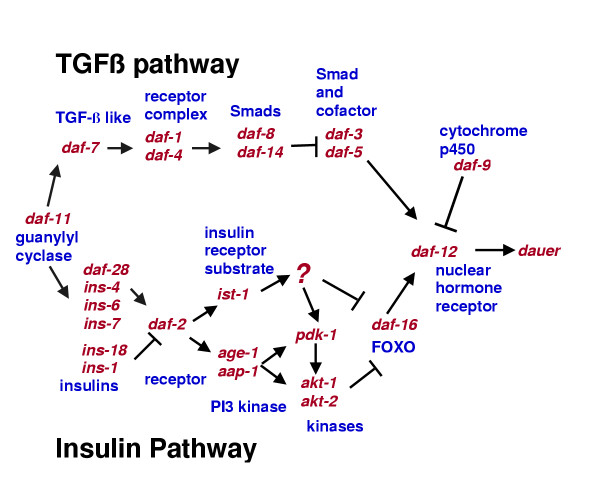
**Signal transduction pathways that regulate dauer. **Relationships between genes are based on mutant phenotypes and genetic interactions, gene expression in mutants, and homology to pathways in other organisms.

Figure 1 shows two pathways, related to TGFβ and insulin/IGF pathways in vertebrates, which repress dauer formation under non-inducing conditions [1-4]. Studies of these signaling pathways have led to some understanding of the basic role of these pathways in regulating dauer formation. Environmental cues received by chemosensory neurons regulate the transcription of genes that encode ligands in each of these pathways:*daf-7 *in the TGFβ pathway and *daf-28 *and perhaps other insulin-like ligands in the insulin/IGF pathway [5-7]. *daf-7 *and *daf-28 *are expressed in chemosensory neurons in the amphid sensillae, which senses environmental cues that regulate dauer formation. Experiments in which components of the TGFβ and insulin/IGF pathways were expressed from tissue-specific promoters suggest that these pathways function predominantly or entirely in the nervous system to control dauer formation [8-10]. Little is known about how transcription factors regulated by these pathways control dauer entry, except that these signaling pathways probably influence a hormonal signal, because most of the cells altered in dauer are not innervated. Two components downstream of these pathways are DAF-12, a nuclear hormone receptor [11], and DAF-9, a cytochrome p450 that is a putative hormone biosynthetic enzyme [12,13]; these two genes suggest that the secondary signal may be a ligand derived from a lipid.

Dauers are long-lived [1], and mutations in many genes, particularly insulin/IGF pathway genes, affect *C. elegans *lifespan [14]. The role of the insulin/IGF pathway in aging appears to be shared by related pathways in Drosophila and mice [15]. Unlike mutations in insulin/IGF pathway genes, mutations in TGFβ pathway genes do not extend adult lifespan.

Many genes have been shown to be regulated by dauer formation and by the insulin/IGF pathway. However, no previous studies have focused on regulation of the decision to enter dauer. In the present study, we have used microarray-based analysis of gene regulation in *C. elegans *TGFβ mutants to identify genes that are directly and indirectly regulated by TGFβ signaling during the dauer formation process. Using a stringent definition for significance, we identified over 1200 genes that are upregulated and downregulated in dauer entry. In this study, we focus on the expression and regulation of genes that encode proteins involved in signal transduction and gene regulation. Analysis of these genes has helped suggest mechanisms by which TGFβ signaling might regulate a secondary hormonal cue and how signaling from TGFβ, insulin/IGF, and other dauer regulators is integrated.

We have identified genes that show different levels of expression in a comparison between wild type L2 or L3 larvae (non dauer), and several TGFβ mutants undergoing dauer larva formation (L2d-early dauer). We identify a large number of genes encoding regulatory proteins that are regulated by TGFβ signaling; of special interest are a large group of putative hormone biosynthetic enzymes and hormone receptors. We see strong regulation of many genes related to Hedgehog and its receptor, Patched; our analysis of genes coregulated with these signaling molecules suggests that the dauer-regulated members of this family function in cuticle synthesis or other hypodermal functions. Genetic evidence shows that inactivation of either the TGFβ pathway or the insulin/IGF pathway is sufficient to induce dauer formation. These results can be explained in on of two ways. First, inactivation of either pathway, even with continued activity of the parallel pathway, causes dauer formation. Second, inactivation of one pathway may lead to an inactivation of the other. We show that, when the TGFβ pathway is inactive, expression of several genes is regulated in such a way as to decrease signaling in the insulin/IGF pathway. Also, expression of the *daf-12 *nuclear hormone receptor gene is strongly upregulated and expression of the *daf-9 *cytochrome p450 gene is downregulated. These results suggest that feedback inactivation of the insulin/IGF pathway and feedback regulation of other genes may be required in order for TGFβ pathway mutants to cause dauer formation. We suggest that this feedback promotes a clear on/off decision for dauer, even under moderate dauer-inducing conditions, and discuss the relevance of feedback for control of aging. We find altered expression of more than 70 genes with putative DAF-16 regulatory sites, and find that whether these genes are upregulated or downregulated depends on the location of the putative DAF-16 site. Finally, we see regulation of many insulin-related genes as well as genes that encode proteins similar to the extracellular domain of insulin/IGF receptors; this variety of signaling molecules may allow insulin/IGF signaling to have a dramatically different outcome in dauers and adults.

## Results

### Array-based hybridization experiments and analysis of expression profiles

We compared gene expression in *C. elegans *wild type to that of animals mutant for the TGFβ pathway genes *daf-7 *(ligand; [5,6]), *daf-8 *and *daf-14 *(Smad transcription factors; [8,16]). At 25°, these *daf-c *mutants form an L2d instead of the L2 larva, and begin dauer morphogenesis after the L2d to dauer molt. Completion of morphogenesis takes about 12 hours from the molt [1]. For all experiments, we examined RNA from animals close to the molt between the L2 (or L2d) and L3 (or dauer). We compared wild-type animals that had just completed the lethargus associated with the L2/L3 molt to *daf-7 *animals that had just completed the lethargus associated with the L2d/dauer molt (four independent experiments). For comparisons of *daf-8 *or *daf-14 *to wild type, we used a slightly earlier stage, and compared wild type animals near the beginning of the L2/L3 lethargus to animals near the beginning of the L2d/dauer lethargus (three independent experiments each).

**Figure 2:**
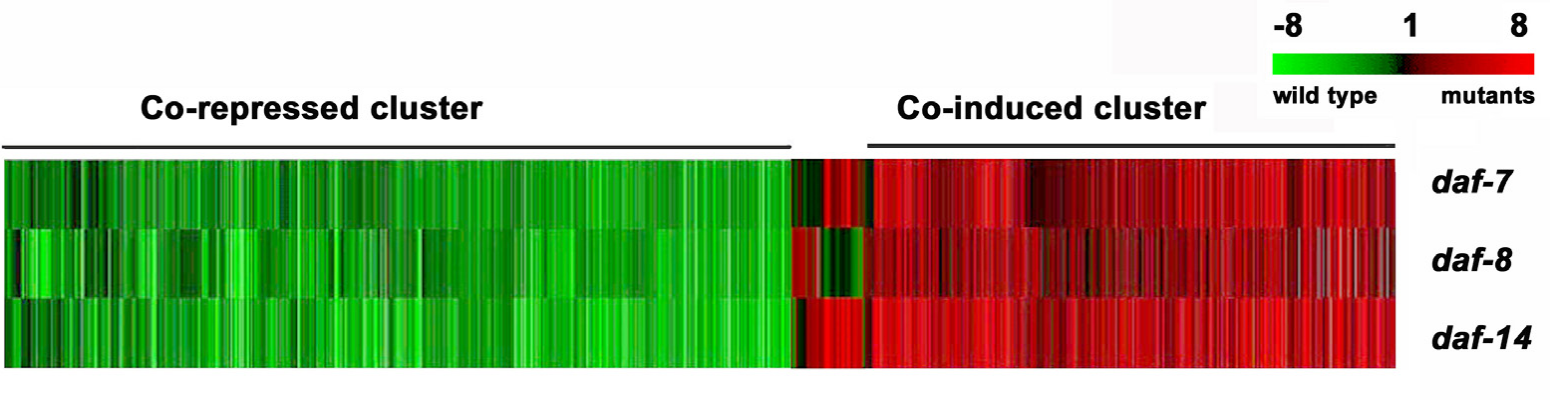
**Cluster diagram of gene expression responses. **Each row represents an average of 3–4 independent experiments. Each gene on the array is represented as a line in each column. The color of the line represents the log2 of the expression ratio, as indicated by the scale bar.

We found that many genes showed significant regulation in at least one genotype (t-test p < 0.05; Table 1; full data in additional file 1: Table S1). More than 90% of the genes showed consistent regulation; if a gene had significantly altered expression in one mutANT, the gene was regulated in the same direction in the other two mutants (Fig. 2). This consistency is not surprising, because all three *daf-c *genes are in the same signal transduction pathway and have similar phenotypes. Because the results from the three *daf-c *genotypes were broadly similar, we analyzed combined data from all three genotypes. By repeating our statistical analysis on the combined data set, we identified 3248 (out of genes that were significantly up- or down-regulated (P < 0.01). We identified over 1200 genes that were strongly regulated (>2.1 fold, p < 0.01; see additional file 2: Table S2).

**Table 1:** Significantly regulated early dauer genes in three TGFβ pathway mutants.

**Genotype**	**Harvest stage**	**Number of experiments**	**# genes up-regulated p < 0.05**^a^	**# genes down-regulated p < 0.05**
*daf-7 *vs. wild type	early dauer/L3	4	351	1069
*daf-8 *vs. wild type	late L2d/L2	3	180	679
*daf-14 *vs. wild type	late L2d/L2	3	552	1276
*daf-c *vs. wild type^b^		10	2540	3076

^a ^Upregulated genes are expressed at a higher level in *daf-c *mutants than in wild type, and downregulated are the reverse.

^**b **^This row is not a summary of the rows above, rather, it is a reanalysis of the data with all ten experiments considered together.

We compared our results to published analysis of gene expression in dauers. Many studies have reported the identification of "dauer-regulated" genes, but this term covers a vast range of very different experiments, and we carefully selected data for validation (see Methods). We found that our data was in good agreement with published data from experiments of similar design (see Methods and Table 2); 7 of 8 genes examined were similarly regulated in our data and published experiments.

**Table 2:** Comparison of microarray data to published experiments.

**Gene**	**Method**	**Published difference**	**Fold change, this study**	**P value**
C27H5.5/*col-36*	RT-PCR	L2d/dauer >> L2/L3^a ^[64]	6.8	<0.008
C47G2.1/*cut-1*	Northern	L2d/dauer >> L2/L3^a ^[65]	5.7	<0.003
C08A9.1/*sod-3*	Northern	present in dauer, absent in non-dauer [66]	7.8	<0.00001
T01B7.7/*rol-6*	Northern and slotblot	L2/L3 >> L2d/dauer^a ^[61]	-7.4	<0.00002
B0491.2/*sqt-1*	Northern and slotblot	L2/L3 >> L2d/dauer^a ^[61]	-7.0	<0.0002
T23G5.1/*rnr-1*	GFP fusion	not seen at L2d/dauer molt, seen in cells in S phase at L2/L3 molt [67]	-1.8	<0.05^a^
W01B6.7/*col-2*	Northern and slotblot	L2d/dauer >> L2/L3^a ^[61]	2.2	<0.02
T13B5.4/*col-40*	RT-PCR	L2d/dauer >> L2/L3^a ^[64]	1.1	<0.8

^a ^L2d/dauer refers to animals near the molt between these two larval stages; L2/L3 also refers to animals near the molt.

^b ^The P value for *rnr-1 *was calculated using only the local standard deviation because the global standard deviation was not available.

### Many strongly regulated early dauer genes have DAF-16 regulatory sites

We examined the regulated genes in our data set to see if we could identify possible cis-acting regulatory sites. Three Smads (DAF-8, DAF-14, and DAF-3) act downstream of the receptors. Two of the Smads do not have an identifiable DNA binding domain [4,5,16], and may serve to negatively regulate the DAF-3 Smad [4,17]. Unfortunately, the optimal binding site for DAF-3 is only 5 bp [18]. Most genes (>80%) have a DAF-3 binding site within 2000 bp upstream of the ATG (data not shown).

Therefore, we focused on the insulin/IGF pathway that regulates DAF-16, which has an eight bp optimal binding site in experiments performed in vitro, TTGTTTAC [19]. We expect that regulated genes in our data set include targets of DAF-16 for three reasons. First, signaling from the insulin/IGF pathway is decreased in the TGFβ pathway mutants (see below). DAF-16 is negatively regulated by DAF-2 signaling, so we would expect that DAF-16 would be more active in our mutant worms. Second, DAF-16 relocalizes to the nucleus when it is active, and *daf-7 *mutants accumulate DAF-16 in the nucleus during dauer entry [20]. Third, DAF-16 and DAF-3 both promote dauer formation, so might have targets in common.

**Figure 3:**
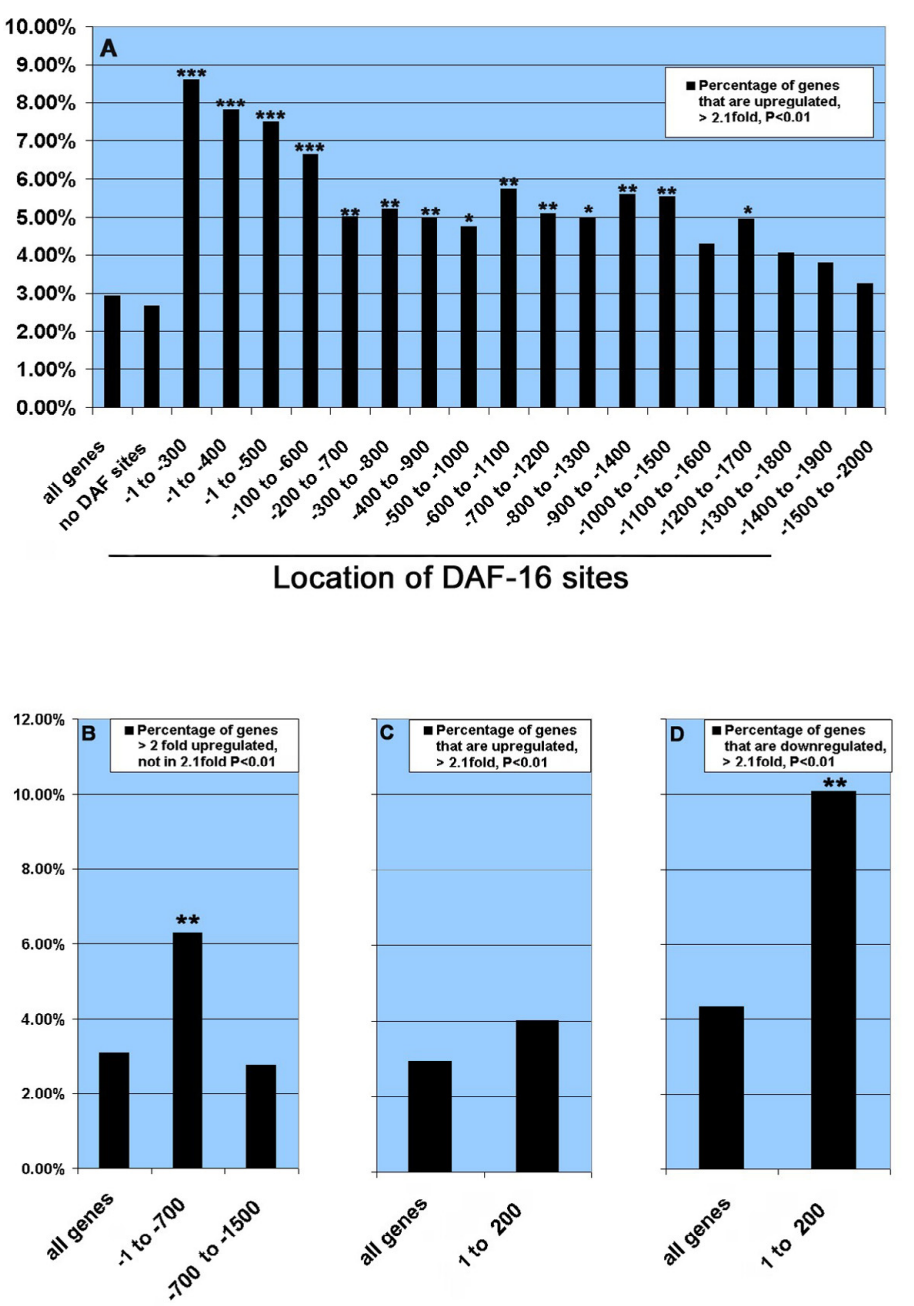
**Regulation of genes with DAF-16 binding sites. **In all panels, the bars show groups defined by the location of DAF-16 sites, as shown on the X-axis. We used overlapping intervals to allow robust statistical analysis. Negative numbers are bp upstream of the initiation codon, and positive numbers are bp downstream of the stop codon. Asterisks indicate statistical significance (exact hypergeometric probability) of the proportion of strongly regulated genes in the group compared to all genes:*** is p < 0.001, ** is p < 0.01, * is p < 0.05. A. Strongly upregulated (>2.1 fold, p < 0.01) genes. B. Two fold upregulated genes, excluding those genes in panel A. C. Strongly upregulated genes with downstream DAF-16 sites. D. Strongly downregulated (<2.1 fold, p < 0.01) genes.

We see strong evidence that genes with DAF-16 sites upstream of transcription are likely to be upregulated. 2.7% of genes without DAF-16 sites are strongly upregulated, but genes with DAF-16 sites in all intervals from -1 to -1500 have significantly more upregulated genes (Fig. 3A, additional file 3: Table S3). 82 genes with a DAF-16 site within 1500 bp upstream of the translation start are strongly upregulated against a random expectation of 40. We also examined genes that did not meet our most stringent criteria for regulation: these genes were at least two-fold upregulated, but not included in the analysis in Fig. 3A. We found 50 genes with a DAF-16 binding site within 700 bp upstream of the translation start against a random expectation of 24 (Fig. 3B; additional file 3: Table S3). We examined 734 strongly downregulated genes, and see no increased likelihood of upstream DAF-16 sites (58 genes expected, 58 observed). When we examined genes with DAF-16 sites within 200 bp downstream of the stop codon, we see no correlation with upregulation (Fig. 3C). However, we see 15 downregulated genes with DAF-16 binding sites against a random expectation of 6 (Fig. 3D, additional file 3: Table S3).

### Insulin/IGF pathway signaling is regulated by feedback from TGFβ signaling

#### Insulin/IGF receptor kinase and other downstream signaling molecules

Because we found significant regulation of genes with DAF-16 binding sites, we examined regulation of genes in the insulin/IGF pathway, which acts to antagonize DAF-16 signaling. Several genes in the insulin/IGF-like pathway that regulates dauer formation (see Fig. 1) are strongly regulated. The most critical of these is *daf-2*, which encodes the insulin/IGF receptor, because, downstream of the receptor, the pathway bifurcates, and mutants in downstream genes have weaker phenotypes [21-23]. Downregulation of *daf-2 *in TGFβ pathway mutants suggests that signaling from the TGFβ pathway positively regulates signaling from the insulin/IGF pathway. Published genetic analysis shows that a nuclear hormone receptor, DAF-12, is antagonized by signaling from the TGFβ receptors [3]. One means of antagonism may be to regulate a hormone that controls DAF-12 activity. A cytochrome p450 gene, *daf-9*, has a partial dauer constitutive mutant phenotype, and acts upstream of *daf-12 *and downstream of the TGFβ pathway to repress dauer formation [12,13]. In dauers, a *daf-*9::*GFP *reporter is downregulated in hypodermis and upregulated in a pair of cells in the head [12]. We see strong downregulation of *daf-9 *in our data; these results are consistent, given that the hypodermis is at least two orders of magnitude larger than the head cells. We also find that *daf-12 *itself is strongly upregulated in our data; this is a form of regulation that had been previously unidentified. Upregulation of *daf-12 *and downregulation of *daf-2 *and *daf-9 *would all be predicted to induce dauer formation.

Other genes in the insulin/IGF pathway are regulated, but the regulation is complex, with both induction and repression. AKT-1, ATK-2 and PDK-1 are kinases that negatively regulate DAF-16, a FOXO transcription factor. A loss of function mutation in *pdk-1 *causes a dauer constitutive phenotype that is much weaker than null mutations in *daf-2 *[23]. Simultaneous knockdown of *akt-1 *and *akt-2 *by RNAi also gives a dauer constitutive phenotype that also is weaker than a *daf-2 *null. Single knockdown of *akt-1 *or *akt-2 *by RNAi does not produce a dauer constitutive phenotype [24]. The weaker phenotype of *akt-1*, *akt-2 *and *pdk-1 *is at least partly due to redundancy in the pathway; a second output from DAF-2 is transduced by IST-1 [21]. The phenotypes may also be weaker because the genes were not completely knocked out by RNAi. We see upregulation of *pdk-1 *and one isoform of *akt-1*, and downregulation of *akt-2*, one isoform of *akt-1*, and some, but not all, isoforms of *daf-16*. The overall effect of this mix of regulation is hard to predict, but suggests variable function for different genes and isoforms. For example, *akt-1a*, *akt-1b *and *akt-2 *may be differently regulated in different tissues, with the result that the output of signal transduction is subtly different.

#### Regulation of insulin/IGF and novel genes with similarity to the extracellular domain of insulin/IGF

*C. elegans *has one insulin/IGF receptor kinase, but dozens of insulin-related genes [24]. The reason for the abundance of ligands is unclear, but perhaps multiple insulin-like ligandss allow for different temporal and spatial patterns of receptor activation, or produce receptor-ligand complexes with different activities. The structure of the insulin-like genes is quite diverse, with three major groups defined by the pattern of disulfide bonds, and subgroups defined by differences in gene structure [24]. Diversity in function of insulin like genes has been demonstrated; INS-4, INS-6, INS-7 and DAF-28 have been shown by genetic or biochemical analysis to be receptor agonists [7,25,26], and INS-1 and INS-18 act as antagonists [24].

We see strong upregulation and downregulation of a subset of these insulin-like genes. Insulin-like genes in *C. elegans *have been divided into three types on the basis of disulphide bonding patterns [24]. These three groups can be further subdivided on the basis of other sequence features. One subgroup has 9 genes, *ins-2 *through *ins-9*, and *daf-28*. Six of these are present on the array, and four (*ins-4*, *ins-5*, *ins-6 *and *ins-7*) show significant downregulation (Table 3). The other two insulin-like genes in this family that are present on the array (*ins-2 *and *ins-3*) are not significantly changed. INS-6 has been shown to be a receptor agonist in vitro [25]. Overexpression of *ins-4 *and *ins-6 *can partially or completely rescue a *daf-28 *mutant [7], and *ins-7 *RNAi enhances a weak *daf-2 *mutant [26], suggesting that these insulin-like ligands act as DAF-2 agonists. Three insulin-like genes are upregulated (Table 3); only one of the three, *ins-18*, has an identified function, and it acts as a receptor antagonist [24]. Increased expression of this ligand, like reduction in expression of *daf-2*, *ins-6*, etc., would be expected to promote dauer formation.

**Table 3:** Dauer and aging genes regulated by TGFβ signaling.

**Gene**	**Induces or represses dauer?**^a^	**Fold regulation**^b^	**p value**
**insulin pathway**			
*daf-2 *(receptor)	strongly represses	-3.1	<0.0001
*akt-2*	represses	-3.0	<0.0001
*akt-1 *all^c^	represses	1.0	ns
*akt-1a*^c^	unknown	-1.8	<0.003
*akt-1b*^c^	unknown	1.4	<0.03
*pdk-1*	represses	1.8	<0.0001
*daf-16a*^d^	unknown	1.0	ns
*daf-16 *all^d^	promotes	-1.7	<0.0004
**Insulin-like ligands**			
*ins-4*	represses^e^	-2.1	<0.002
*ins-5*	unknown	-2.6	<0.02
*ins-6*	represses^e^	-2.6	<0.02
*ins-7*	represses	-2.1	<0.005
*ins-18*	promotes^e^	1.8	<0.002
*ins-33*	unknown	3.9	<0.0003
*ins-35*	unknown	6.5	<0.0001
**insulin receptor-like**			
F56A4.C	unknown	-3.5	<0.0001
Y19D10A.7	unknown	-2.8	<0.0003
F14D2.6	unknown	1.8	<0.006
F15E11.11	unknown	1.8	<0.008
**other dauer and aging regulators**			
*daf-12*	strongly induces	2.2	<0.0007
*daf-5*	induces	-2.0	<0.0003
*daf-9*	represses	-4.2	<0.02
*scl-1*	unknown	5.2	<0.006

ns, not significant This

table shows all insulin-like ligands, insulin-receptor-like genes, TGFβ, and insulin/IGF pathway genes with greater than 1.8-fold regulation and a p value less than 0.05. Genes checked for regulation that showed <1.8 fold regulation or p > 0.05 or both: *clk-1*, *clk-2*, *sir-2.1*, *daf-11*, *daf-7*, *daf-1*, *daf-4*, *daf-14*, *daf-8*, *daf-3*, *daf-19*, *tax-4*, *npc-1*, *daf-18*, *age-1*, *old-1*, *mev-1*, *hsf-1*, *daf-21*, *gpa-2*, *gpa-3*, *kin-29*, *kin-8*, *unc-3 *and *tph-1*, as well as all genes annotated as insulins present on the microarray (*ins-2*, *ins-3*, *ins-11*, *ins-17*, *ins-22*, *ins-23*, *ins-24*, *ins-26*, *ins-30*, *ins-32*, *ins-34*, *ins-37*) and nearly all genes identified as putative insulin receptors in Dlakic [27].

^a^Induction or repression of dauer is defined by mutant or RNAi loss-of-function phenotypes unless otherwise noted. *daf-9 *mutants have characteristics similar to both types, and are thus marked "mixed".

^c^The fold change between mutant dauers and wild-type. A positive number indicates higher expression in dauers, a negative number indicates higher expression in N2.

^c^The table entry "*akt-1 *all" is for a probe that recognizes both isoforms. The other two *akt-1 *entries each cover an exon unique to the indicated isoform.

^d^The entry "*daf-16a *isoform" is for a probe that recognizes:R13H8.1c (*daf-16a1*), R13H8.1b (*daf-16a2*), and R13H8.1d, but not R13H8.1a (*daf-16b*) or R13H8.1e. The entry "*daf-16 *all" is for a probe that recognizes all five isoforms of *daf-16*.

^e^Overexpression of *ins-4 *or *ins-6 *represses dauer in a *daf-28 *mutant [7]. *ins-6 *has also been shown biochemically to act as a receptor agonist [25]. Overexpression of *ins-18 *promotes dauer [24].

A family of genes with modest but significant similarity to the ligand binding domain of insulin/IGF receptor has been identified [27], and these may contribute to insulin/IGF signaling and signaling diversity; however, because of weak similarity to insulin/IGF receptor and similarity to other receptor tyrosine kinase ligand binding domains, a function in insulin/IGF signaling is speculative. As with the insulin-like genes, we see both upregulation and downregulation of insulin-receptor like molecules.

### Regulation of regulatory genes

Genetic and molecular analysis has allowed us to gain a basic understanding of the key regulatory pathways that affect dauer formation by acting in the nervous system, but we know little about regulatory events downstream of these pathways, i.e. the regulators that actually control dauer morphogenesis and physiology. Below, we discuss a selected set of regulated genes; a complete list of regulatory genes can be found in additional file 4: Table S4.

#### Regulators of aging

Because dauers are long lived, and because many genes that control lifespan also control dauer entry, we examined the expression of genes with mutant phenotypes of long or short lifespan. Other than the insulin/IGF pathway genes mentioned above, *scl-1 *was the only aging regulatory gene we identified as strongly regulated (Table 3). SCL-1 has an SCP domain, which defines a family of putative signaling molecules with unknown biochemical function [28,29]. The *scl-1 *gene is required for extension of lifespan in a *daf-2 *mutant, and *scl-1 *expression is upregulated in long-lived genotypes [28]. *scl-1 *is strongly upregulated in our data as well, and we suggest *scl-1 *is part of the mechanism of lifespan extension in dauers. Several other genes in the *scl-1 *family are upregulated in old animals and in mature dauers, but not in our data [29,30,52].

#### Cytochrome p450s

Cytochrome p450s are versatile enzymes that oxygenate a wide variety of compounds [31]. These reactions are involved in protection from toxins, hormone biosynthesis and other functions. Cytochrome p450s are very prominent among our regulated genes (additional file 4: Table S4); 43 of 75 are regulated >1.8 fold (p < 0.05). One putative hormone biosynthetic cytochrome p450, *daf-9*, is known to function in dauer formation, and these others may also participate in hormone metabolism in dauer.

#### Hedgehog/Patched

In *Drosophila *and vertebrates, Patched and Smoothened form a receptor complex that binds Hedgehog ligands. *C. elegans *has two functional Patched orthologs and a family of proteins similar to Patched, called Patched-related. One, *ptc-1*, has an RNAi phenotype of defective cytokinesis in the germ line [32]. However, none of the *patched/hedgehog *family genes shown in Table 4 has an RNAi phenotype similar to *ptc-1 *[51,33]. A group of 10 *C. elegans *proteins have a carboxyl terminus that is related to the carboxyl terminus of Hedgehog; however, no protein with substantial similarity to the N-terminal signaling domain of Hedgehog has been found in *C. elegans*. Instead the *C. elegans *proteins have two novel families of N terminal sequences, called Wart and Ground domains [34]. Genes that encode both the N-terminal domain and the Hedgehog-related C terminal domain are called *warthog *and *groundhog*. Some proteins have only Wart or Ground domains. Finally, a large family of proteins has a domain that is similar to the Ground domain, and these are called Ground-like. Careful examination of the sequence indicates that Wart, Ground, and Ground-like domains and the N terminal domain of Hedgehog, despite low sequence identity, share motifs that indicate descent from a common ancestor [34].

**Table 4:** Regulatory genes regulated by TGFβ signaling.

**Hedgehog and Patched**				
Gene	Type of gene	Fold regulation	p value	Mountain
*grd-2*	groundhog	-2.4	0.0005	14
*grd-7*	ground domain only	3.8	0.0003	13
*grd-6*	ground domain only	-3.1	0.0002	16
*grd-14*	ground domain only	-8.9	0.0002	16
*wrt-7*	warthog	6.7	0.01	17
*wrt-1*	warthog	-6.8	0.0001	14
*wrt-4*	warthog	-2.9	0.0003	14
*wrt-6*	warthog	-2.7	0.0005	14
*wrt-8*	warthog	-3.8	0.0005	14
*wrt-2*	wart domain only	-1.8	0.008	14
C56A3.1	ground-like	4.8	0.0001	17
K03B8.7	ground-like	18.9	0.0003	17
ZC487.4	ground-like	3.4	0.009	17
C24G6.7	ground-like	-3.6	0.0006	14
F42C5.7	ground-like	-2.4	0.0007	1
T02E9.2	ground-like	-7.7	0.0001	14
Y75B8A.20	ground-like	-13.3	0.0001	14
ZC168.5	ground-like	-4.6	0.0001	16
*ptc-3*	patched	-2.1	0.0001	1
*ptr-6*	patched related	-2.3	0.0008	14
*ptr-11*	patched related	-2.6	0.0002	1
*ptr-16*	patched related	-2.7	0.01	14
*ptr-18*	patched related	-3.3	0.0002	16
*ptr-23*	patched related	-2.5	0.0003	6
*npc-2*	patched family	-2.5	0.0004	16
**Dauer growth arrest**				
Gene	Type of gene	Fold regulation	p value	
*dbl-1*	TGFβ ligand, regulates growth and body size	-2.4	0.001	
*cdk-4*	cyclin dependent kinase	-1.9	0.006	
*nhr-73*	member of a family of NHR genes expressed exclusively in lateral hypodermis (seam cells)	-4.4	0.00002	
*nhr-74*	member of a family of NHR genes expressed exclusively in lateral hypodermis (seam cells)	-5.5	0.00005	
*nhr-25*	similar to NHR in Drosophila ecdysone regulatory cascade, required for embryogenesis and molting	-2.4	0.001	
**G-protein-coupled receptors and olfaction**				
gene	type of gene	fold regulation	p value	
*srh-75*	chemoreceptor	2.6	0.007	
*srh-195*	chemoreceptor	2.6	0.0009	
*srj-32*	chemoreceptor	5.8	0.002	
*sru-21*	chemoreceptor	6.3	0.0007	
32 genes	chemoreceptor	1.8 to 6.3	0.05 or less	
15 genes	GPCR, not chemoreceptor type	1.8 to 3.6	0.05 or less	
C30F12.6	thyrotropin-releasing hormone receptor ortholog	1.8	0.002	
*npp-10*	GPCR, nucleoporin	-1.8	0.001	
6 genes	chemoreceptors	-1.8 to -3.8	0.05 or less	
*gpa-10*	G protein alpha subunit	2.0	0.003	
Y71H2B.7	G protein alpha subunit	-1.8	0.0001	
F45B8.2	regulator of G-protein signaling domain	2.4	0.03	
*gcy-22*	receptor guanylate cyclase	2.7	0.03	
*gcy-31*	soluble guanylate cyclase	1.8	0.01	
*gcy-34*	soluble guanylate cyclase	1.8	0.03	
*tax-2*	cyclic nucleotide gated channel beta subunit	3.0	0.02	
*lim-6*	homeobox protein, functions to allow chemosensory neurons to sense different molecules	2.0	0.003	
**Notch pathway**				
gene	type of gene	fold regulation	p value	
W02C12.1	notch family	3.9	0.0007	
F47C12.1	notch family	7.4	0.0003	
*apx-1*	ligand for GLP-1, notch receptor	1.8	0.02	
R03D7.5	shaggy/GSK3 kinase	2.2	0.002	
*lag-1*	ortholog of CBF1 and Suppressor of Hairless	1.6	0.0003	
**Transcriptional regulators**				
gene	type of gene	fold regulation	p value	
*lin-28*	cold-shock domain	1.8	0.0001	
*lin-29*	zinc finger transcription factor	-1.9	0.0002	

A complete list of significantly regulated regulatory genes can be found in additional file 4: Table S4.

We see abundant regulation of these gene families in our data (Table 4). Both upregulated and downregulated genes are observed, but downregulated genes are much more abundant.

#### Olfaction and other G-protein-coupled receptor signaling

Two of the three well-characterized cues for dauer entry are food and pheromone, which are sensed by chemosensory neurons. Chemosensation in *C. elegans *is mediated by G protein coupled receptors (GPCRs), which regulate cyclic GMP levels [35]. We see many upregulated G protein coupled receptors, accessory proteins, and other proteins related to olfaction, but few downregulated genes of these types (Table 4).

#### Cell cycle and growth arrest

Many cell types undergo cell cycle arrest in dauers. We see downregulation of *cdk-4 *(homologous to human *Cdk-4/Cdk-6*), which is well suited to control cell cycle arrest in dauers. The *cyd-1 *(cyclin D) and *cdk-4 *genes function in larval development to promote exit from the G1 stage of the cell cycle [36]. Animals with loss of function of either gene fail to carry out postembryonic divisions. We see a significant reduction in expression of *cdk-4 *(Table 4), which would be predicted to cause cells that would normally divide in reproductive L3 to arrest in dauers. However, loss of *cdk-4 *would not be expected to lead to an arrest of all growth in dauers. Some cells in *C. elegans *have endoreduplication without cell division, producing large hyperploid cells. DBL-1 is a ligand in a TGFβ pathway that controls larval growth [4,37]. *dbl-1 *mutants grow less than wild type, and have lesser DNA content in hyperploid hypodermal cells [38]. We see downregulation of *dbl-1 *in dauers (Table 4), which may block endoreduplication, and thereby arrest growth, in cell types such as intestine and hypodermis. Thus the microarray analysis suggests a model for the arrest of both cell division and endoreduplication.

#### Notch

Notch pathways have not been previously implicated in dauer formation, but we see strong evidence for altered activity of these pathways in dauer entry. Two of nine genes annotated as Notch receptors in *C. elegans *show strong upregulation in our data, as does one ligand, *apx-1*. We also see regulation of two genes that act downstream of the receptors: a shaggy/GSK3 kinase and *lag-1*, a transcription factor [20]. The regulation of these various Notch pathway components suggests that Notch signaling has an important unidentified function in dauer formation.

#### lin-28 and lin-29

These two genes are part of a signal transduction cascade that regulates timing of developmental events during larval development [39]. During larval growth, abundance of the cold shock domain containing LIN-28 protein is post transcriptionally downregulated. This downregulation allows the zinc-finger transcription factor LIN-29 to accumulate and promote events appropriate to the third larval stage. In our data, *lin-28 *is upregulated, and *lin-29 *downregulated (as expected, since *lin-28 *negatively regulates *lin-29*). This regulation is consistent with some old observations of the role of these genes in dauer formation [40]. *lin-28 *loss-of-function mutants enter dauer, but have defects in dauer morphogenesis. These defects are suppressed by *lin-29 *mutations. The regulation we see is consistent with the prediction that downregulation of *lin-29 *by *lin-28 *is required for normal dauer morphogenesis [40].

## Discussion

### Feedback model for crosstalk between TGFβ pathway and other dauer regulatory genes

We find that mutations in TGFβ signaling genes affects the expression of numerous genes that are known regulators of dauer formation. For example, loss of TGFβ signaling causes reduction of expression of *daf-2*, would be expected to reduce signaling through the insulin/IGF pathway. In addition, the TGFβ pathway mutants have increased expression of *daf-12 *and reduced expression of *daf-9*, a cytochrome p450 that is predicted to be involved in the biosynthesis of a *daf-12 *antagonist. All of these changes in gene expression would be predicted to promote dauer formation, and suggest that one way that the wild-type TGFβ pathway promotes reproductive growth is by feedback regulation of other dauer pathway genes.

We propose that this regulation is part of a feedback mechanism that operates at many levels to insure a "clean" dauer/non-dauer decision. Under conditions that induce dauer formation to different extents, different percentages of the population form dauers, but individual animals either undergo morphologically complete dauer, or have no dauer morphogenesis; therefore, a mechanism must exist to convert ambiguous signals into a clear decision. If reduced TGFβ signaling causes a reduction in insulin/IGF signaling and vice versa, then weak signals can be strengthened to make a clear, organism-wide decision. Negative regulation of *daf-12 *and upregulation of *daf-9 *by TGFβ and insulin/IGF signaling would likewise amplify signals. Interestingly, *daf-2 *and *daf-12 *both function as "nodes" for signal transduction in dauer entry. Many genes function in TGFβ and insulin/IGF pathways in dauer formation, but all of them except *daf-2 *show genetic redundancy [1,3,8,21-23,41]. Regulation of genes other than *daf-2 *would produce a weaker effect on dauer formation. Similarly, *daf-12 *is the gene that TGFβ and insulin/IGF signaling converge on, and regulation of *daf-12 *would be expected to have a uniquely powerful effect on dauer formation.

The feedback regulation to the insulin/IGF pathway explains published observations that link the TGFβ pathway to the insulin/IGF pathway. First, *daf-16 *mutants partially suppress *daf-7 *and other mutants in the TGFβ pathway [3]. We suggest that feedback from the TGFβ pathway to the insulin/IGF pathway is essential for complete dauer formation. In this model, loss of *daf-16 *function does not have a direct effect on TGFβ signaling, rather, *daf-16 *mutants suppress the inactivation of insulin/IGF signaling that occurs in the TGFβ mutants. Second, DAF-16 is localized to the nucleus when insulin/IGF signaling is weak, for example in *daf-2 *mutants of all ages. DAF-16 is localized to the nucleus in *daf-7 *mutants that are entering dauer, but not at other stages [20]. We suggest that this localization is a consequence of feedback regulation from the TGFβ pathway to the insulin/IGF pathway. Third, insulin/IGF pathway mutant adults have a variety of stress-resistance phenotypes, including slowed aging, which are shared with dauers, but not with TGFβ pathway mutant adults. We suggest that the stress resistance seen in *daf-7 *dauers is mostly or entirely caused by feedback regulation of the insulin/IGF pathway, and that this feedback occurs in *daf-7 *mutant dauers, but not at other stages.

### Regulation of insulin-like ligands and novel genes with similarity to insulin/IGF receptors suggests functions for these genes in dauer formation

We find several insulin-related genes and genes with similarity to insulin receptor that are regulated by TGFβ signaling. Four insulin-like genes are downregulated; these are members of a subfamily in C. elegans that encodes several agonists of insulin/IGF signaling. Published data suggest that three of the downregulated insulin-like ligands act as DAF-2 (receptor kinase) agonists [7,25,26]. Thus, downregulation of these genes and perhaps the closely related gene *ins-5 *would be predicted to promote dauer entry, and may be part of the feedback mechanism proposed above. Published data suggest that ins-18, which is upregulated in our data, acts as a DAF-2 antagonist; [24] upregulation of this gene would be predicted to promote dauer entry, and this gene is another candidate for participating in the feedback mechanism proposed above. Upregulation of *ins-33 *and *ins-35 *suggests that these genes, like *ins-18*, encode receptor antagonists.

The *ins-7 *and *ins-18 *genes, but not the other insulin-like genes in Table 3, are regulated by insulin/IGF signaling in adults. Conversely, several insulin-like genes that are not regulated in dauer entry are regulated by insulin/IGF signaling in adults [26,42]. The gene programs induced by in dauers have similarities to those induced in long-lived adults; for example, stress resistance genes are common to both. However, many gene regulatory programs are unique to dauer, for example growth arrest. The different spectrum of insulin-like genes expressed in dauer entry versus adults may explain at least part of the difference in events regulated by insulin/IGF signaling. As with the insulin-like genes, we see both upregulation and downregulation of insulin-receptor like molecules; this regulation identifies these genes as good targets for studies to determine if this gene family is involved in insulin/IGF pathway signal transduction. These results suggest that diversity in insulin-related ligands, and perhaps in receptors, contributes the ability of insulin/IGF signaling to produce dramatically different phenotypes in dauers and adults

### Many strongly regulated early dauer genes have DAF-16 regulatory sites

We observed that genes with putative *daf-16 *binding sites show a tendency to be upregulated in our data, with the exception of a small number of genes with *daf-16 *sites downstream of the transcription start. These results are important in several ways. Most significantly, our data identify genes that may be directly regulated by DAF-16 to control dauer formation or to provide dauer-specific functions such as protection from environmental insults. Second, genetics predicts that DAF-16 might upregulate genes that promote dauer entry or downregulate genes that promote reproductive growth. Our results suggest that DAF-16 can do both, but upregulated genes are much more prominent in our data set. Third, we see that the direction of regulation is correlated with the position of the DAF-16 site.

Our results show interesting similarities and differences with recent reports about DAF-16 target genes [53,54,42,43]. A significant number of genes that are upregulated in our data are also upregulated in these other experiments, but some genes are differently regulated. These papers all examined gene expression in adults, and in insulin/IGF pathway mutants, whereas our experiments have perturbed but not mutated insulin/IGF signaling (see below), so we would expect that only a subset of genes would be in common. The most dramatic difference between our data and these published reports is that we see evidence for upregulation of genes by DAF-16 sites upstream of the transcription start, but not downregulation, whereas the other reports see as many upregulated genes as downregulated genes. These differences suggest that the activity of DAF-16 is fundamentally different in dauers and adults, presumably because of availability of different cofactors.

### Other regulatory genes

#### Cytochrome p450s

A very large number of cytochrome p450s show strong regulation in our data. One putative hormone biosynthetic cytochrome p450, daf-9, is described above, and these others may also participate in hormone metabolism. Other microarray experiments have identified cytochrome p450s as regulated genes in dauer or under the control of insulin/IGF signaling in *C. elegans *[25,42,52]. Because of the diversity of functions of cytochrome p450s, and especially because of the prominent roles of cytochrome p450 in stress resistance, sorting out the function of these genes in defense and hormone biosynthesis will require detailed functional analysis.

#### Hedgehog/Patched

The function of these genes is uncertain, as most have not been studied. Based on the expression pattern of several Ground and Wart domain proteins, Aspock *et al*.[34] suggested a function for these genes in hypodermis. A comparison of regulated Hog and Patched genes to groups ("mountains") of putatively coregulated genes in *C. elegans *[44] shows that three mountains are highly enriched for these genes. Mount 14 and Mount 16, are enriched for downregulated genes, and Mount 17 is enriched for upregulated genes (Table 4). These mountains are even more highly enriched for *hog/patched *genes that are strongly regulated in our experiments. These three mountains have less than 5% of the genes in *C. elegans*, but 48% of *hog/patched *genes and 80% of *hog/patched *genes that are strongly regulated in our data. All three mountains are also enriched for cuticular collagens; thus, we suggest that the *hog *and *patched *genes function in the production of the cuticle by the hypodermis. The fact that we see substantial numbers of both upregulated and downregulated genes supports this hypothesis. Dauers and non-dauers each form a cuticle, but with dramatically different structures [1]; therefore, we would expect to see the same types of genes in each program, just as we do when examining expression of cuticle structural genes such as collagens and cuticlins (Table 2 and data not shown).

#### Olfaction and other G-protein-coupled receptor signaling

We see regulation of many genes that are candidate olfactory and gustatory signal transduction molecules. This regulation may be involved in changes in response to odor and taste cues in dauers. We see many more upregulated genes in this class than downregulated genes. However, both dauer and non-dauer larvae are responsive to chemical cues, and published results of GPCRs with dauer-regulated changes in expression of reporters are not noticeably biased toward upregulation [45,46]. Perhaps anatomical changes can explain the bias toward upregulation. The key sensillum for regulation of dauer formation is the amphid. In dauers, the amphid pore is filled with an unknown material. In addition, ASI and ASG, which are members of the subset of amphid neurons with a known role in dauer formation, have shortened nerve endings that are more distant from the pore [1]. These changes insulate the neurons from the environment, to an extent. For example, lipophilic dyes such as DiI are taken up by amphid neurons in all stages of *C. elegans *development, except in dauers [45,46]. However, dauers respond readily to chemical cues that regulate dauer formation, which are thought to be sensed by amphid neurons. Perhaps genes that are required for chemosensation are more strongly induced in dauers in order to compensate for reduced signals due to the reduced exposure of the sensory endings.

## Conclusions

In summary, perhaps the most striking conclusions from study are that, in TGFβ pathway mutants, we see altered expression of many genes that are known regulators of dauer formation, and that regulation of DAF-16 target genes depends on the location of the DAF-16 binding site. We see that loss of TGFβ signaling causes changes in gene expression that would be expected to reduce signaling through the insulin pathway. In addition, in TGFβ mutants, we see increased expression of *daf-12 *and reduced expression of *daf-9*, a cytochrome p450 that is predicted to be involved in the biosynthesis of a *daf-12 *antagonist. We also see changes in expression of several insulin-related genes and putative non-kinase insulin receptors. This result suggests that diversity in insulin-related ligands, and perhaps in receptors, contributes the ability of insulin signaling to produce dramatically different phenotypes in dauers and adults. We see evidence that DAF-16 binding sites upstream of the coding region can promote upregulation, but not downregulation of gene expression.

**Figure 4:**
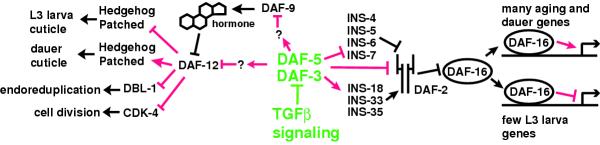
**Model for gene regulatory events under the control of TGFβ signaling. **Transcriptional regulatory events suggested by expression data in this paper are indicated in red. The TGFβ pathway is shown in green; DAF-3 and DAF-5 form a transcription factor complex that function in neurons to control dauer entry [9]. DAF-3 and DAF-5 are shown regulating DAF-9 and DAF-12 indirectly because these genes are likely to be regulated in non-neuronal tissues. Insulin genes are shown as directly regulated by DAF-3 and DAF-5, but it is equally likely that these genes are regulated by feedback from the insulin pathway or by DAF-12.

We identify many other regulatory genes that have altered expression in TGFβ mutants. We see several genes likely to function in Notch pathway signaling; this is the first implication of Notch signaling in dauer formation. We find that many divergent C. elegans homologs of *hunchback *and *patched *are regulated by TGFβ. Because these genes are coregulated with genes that encode structural components of the cuticle, we suggest that these genes regulate the production of the dramatically different cuticles of dauer and reproductive L3. We see coordinate regulation of genes that are predicted to arrest cell division and endoreduplication, which together would be expected to promote growth arrest. We identify many genes that are candidate hormone biosynthetic enzymes that might help transduce signals from the nervous system to other tissues. Figure 4 is a graphic summary of the main conclusions of this work, with new regulatory relationships suggested by our data shown in red.

## Methods

### RNA Isolation, cDNA synthesis, and microarray hybridization

Wild type is *C. elegans *variety Bristol, Strain N2; Daf-c genotypes are *daf-7(e1372)*, *daf-8(e1393)*, and *daf-14(m77)*. Temperature-sensitive strains were maintained at 15°. Eggs were collected by hypochlorite treatment and incubated overnight at 15°. L1 larvae were grown on plates at 25°. During *C. elegans *larval development, each molt is accompanied by a brief period of lethargus when the animal stops pharyngeal pumping, and sheds its old cuticle. Cessation of pumping was scored according to [47] and used to judge the stage of worms for harvesting. For comparing *daf-7 *to N2, worms were harvested at early L3 stage for N2, when >95% of the worm had entered the L3 stage and *daf-7 *at early dauer stage when > 85% of the worm had passed the L2d to dauer molt. Synchronization of development in *C. elegans *populations is imperfect. For *daf-8*/N2 and *daf-14*/N2 experiments, a small sample plate was started 2 hours ahead of the harvest plates. The worms were harvested at late L2 stages for N2 when the worm on sample plate showed maximal cessation of pumping and the very first animals on the harvesting plates entered lethargus. *daf-8 *and *daf-14 *animals were harvested at late L2d stage when 50% worm on the sample plates stopped pumping and the very first animals on the harvesting plates entered lethargus. These animals were not perfectly synchronous, but our monitoring of pumping suggests that the vast majority of worms fall within a four-hour developmental window. Worms were harvested quickly to avoid changes in mRNA levels caused by the collection procedure; no more than 15 minutes elapsed between the start of harvest and addition of Trizol. Each experiment compared RNA samples from the wild-type N2 to Daf-c genotype worms. For each Daf-c genotype, we did three or four independent experiments. RNA was prepared as described previously [48]. Labeled cDNA probe for DNA microarray hybridization was made from 5 μg of poly (A)^+ ^as described [49]. The two cDNA probes were simultaneously hybridized to a single DNA microarray.

### Imaging and data analysis

Arrays were scanned using Axon scanner as described previously [49], collecting measurements for Cy5 and Cy3 separately. The average level of regulation for each gene for each genotype was calculated as the mean of the Cy5/Cy3 ratios. Genes with significant regulation were identified by using the standard deviation from the ten experiments presented in this paper ("local standard deviation") and the standard deviation derived from a several hundred microarray experiments of various types ("global standard deviation"), as described [50]. In most cases, genes were considered significantly regulated only if they were significant in a Student's t test using both measurements of standard deviation. However, for a few hundred genes, global standard deviation was unavailable, and significance was measured using the local standard deviation. Our processed, annotated data are available in additional file 1: Table S1. Raw data compliant with the "Minimum information about a microarray experiment" (MIAME) standard is available at: .

### Analysis of duplicated and reannotated spots

All spots on the array were generated by PCR of genomic DNA, as described [49]. Some PCR reactions were spotted in two separate locations on the array. In this case, both data points were used to calculate the average regulation. The PCR primers were designed a few years ago, and in some cases, EST data or other information has revealed that predictions upon which the primers were based were in error [51]. Three types of error account for the vast majority: 1) PCR products that are no longer believed to amplify sequence from a gene. These spots are excluded from the analysis. 2) Cases in which two PCR products that were thought to represent two different genes actually represent different parts of a single gene. In these cases, each spot was initially analyzed independently, but, when we count the number of genes with a particular property, the genes are not double counted. 3) Cases in which a single PCR product is now believed to overlap two genes. Data from these spots were excluded from the analysis. Fortunately, this type of error was uncommon.

### Cross hybridization

Many genes in *C. elegans *have enough mRNA sequence similarity that our labeled cDNA probes might hybridize to homologous genes, obscuring true patterns of gene regulation. We examined our data and published data to evaluate how widespread this problem is. As a crude measure, we compared pairs of homologous genes and asked whether the genes ever show opposite regulation. Our expectation is that, if cross-hybridization is strong, the two genes will show similar regulation. That is, if one member of a pair shows upregulation in dauers and the other member shows down-regulation, then cross-hybridization is not at a high enough level to obscure true regulation. Of course, the converse is not necessarily true: if two homologous genes show similar regulation, that does not imply that cross hybridization is occurring.

We examined 8 groups of genes using blast to identify the most similar genes. In this way, each of the eight genes was matched with a small group of similar genes (6 were collagens, one was cuticlin, and one was superoxide dismutase). In seven groups, the match was approximately 80% identity over 200–700 bp, and several groups had short stretches (<100 bp) of 90% identical sequence. In each group, significantly different regulation was seen in the data collected for this paper. The last group had matches of >90% over 500 bases or more, and this group showed strong correlation in our experiments.

This analysis defines the limits of specificity. For genes with identities of <80%, and genes with short stretches of 90% identity, cross hybridization is not strong enough to obscure differences between genes. When similarity goes above 90% for more than 100 bp, genes show strongly correlated expression, and these results should be interpreted with caution. Our examination of the collagen, cytochrome p450, glutathione S-transferase, heat shock, and peroxidase, insulin ligand, and insulin receptor families of *C. elegans *indicates that no more than 10% of genes in families have similarity great enough to be an issue. Of course, this number applies to genes in families, and the bulk of *C. elegans *genes will have less similarity.

### Validation of the DNA microarray results

We examined our data to see if it was consistent with published data on dauer gene regulation obtained by Northern blot and reporter gene constructs. The term "dauer-regulated" has been used in many published studies to describe results from experiments that are only superficially similar. It would be inappropriate and misleading to compare our data to most published data on "dauer-regulated" genes. For example, Wang, et al. [52] identified dauer-regulated genes, but they were examining wild-type animals over time (rather than comparing mutants to wild type) and studying the process of exiting dauer (these animals were several days older than the animals we were studying. If we were to use this data to validate our data, we would be making the assumption that the set of genes that are regulated during dauer entry are similar to the set of genes that are regulated in dauer exit. There is no data to support this assumption. Comparison of our data set to the Wang et al. [52] would be appropriate to identify interesting similarities and differences between dauer entry and exit, but to use one data set to validate the other would be an error.

Most experiments that we consider inappropriate for use in validation have one or both of the following characteristics:

1) In our experiments and published experiments summarized in Table 2, animals from which RNA is collected are of similar age, either dauers near the L2d/dauer molt or non-dauers near the L2/L3 molt. Many experiments compare dauer RNA to that of mixed stage worms or adult worms [53-55]. which is not useful for our goal of comparing the L3 to the dauer alternative. For example, using mixed stage RNA, a gene that is expressed at a high level in adults but is otherwise off, would be expected to show expression in a mixed-stage RNA prep, but not in dauer RNA. Yet a gene of this sort does not have a meaningful relationship to the dauer/non-dauer decision or dauer morphogenesis. Similarly, a gene that is expressed equally in dauers and in non-dauer L3 animals would be diluted by RNA from other stages, and therefore, less abundant, in the mixed stage RNA than in the dauer RNA, but the gene is not in fact more abundant in dauers than the non-dauer alternative.

2) Many studies use dauers ranging from about 15 hours to 7 days past the L2d/dauer molt [52,54-59]. Some of these studies also compare starvation-induced dauers to well-fed animals. These studies are often aimed toward understanding issues related to survival of dauers and aging. We would not expect that these studies would necessarily find the same genes to be regulated, because of differences in timing and differences in physiology due to starvation. In particular, we are looking at the very beginning of dauer morphogenesis, so would expect to see genes that are necessary for dauer formation, while the other studies are looking after the completion of dauer morphogenesis, so would expect to see genes necessary for dauer maintenance and survival.

We identified the small subset of experiments that are appropriate for validation of our data set, by identifying experiments that: compare animals close to the L2/L3 and L2d/dauer molts. Table 2 shows that our data agrees very well with published experiments of similar design. The first five genes show strong regulation in the published experiments, and 7–8-fold regulation in our experiments. The next two genes, *rnr-1*, and *col-2*, are also regulated similarly in our experiment and published data, although the magnitude and statistical significance are not as great as for the first 5 genes. The final gene, *col-40*, shows strong upregulation at the L2d-dauer molt in published experiments, but in our data does not show regulation. One possible explanation for this discrepancy is that this gene has been shown to have very rapid induction and repression near the time of molting [60,61]. Thus, seeing regulation of *col-40 *may depend on precisely when the RNA is collected. This gene showed very high variation in mutant/wildtype ratio from experiment to experiment, higher than 99% of the genes on the array. This unusual variation is consistent with the idea that timing is critical for measuring regulation of this gene. A second possible explanation is that the regulation of these genes is obscured by cross-hybridization to other collagen genes; however, the similarity of *col-2 *and *col-40 *to other genes at the level of coding DNA is low enough that we do not expect cross-hybridization to be a serious problem (see previous section of Methods). Overall, seven of eight genes show strong correlation with published data.

### Identification of DAF-3 and DAF-16 binding sites

Binding sites for transcription factors were identified using RSA tools [62,63].

## Authors's contributions

TL participated in the design of the study, performed wet lab experiments, analyzed data, and co-wrote the manuscript. KKZ participated in the growth and collection of samples for RNA. GIP conceived of the study, participated in the design and data analysis, and co-wrote the manuscript. All authors read and approved the final manuscript.

## Supplementary Material

Additional File 1 Full data set. Table S1 may be found online as an Excel file at:  Please feel free to use any annotation in these tables, provided that the original source of the data is cited, and this collection of annotations is cited. Table S1 has the data for all of the genes on the microarray. Raw data in unprocessed, MIAME compliant format is available at: . Column A, "SMD gene name" is the name given to the probe on the microarray. The probe is a PCR product from a pair of primers made to amplify the ORF named in this column (Reinke, V., Smith, H. E., Nance, J., Wang, J., Van Doren, C., Begley, R., Jones, S. J. M., Davis, E., Scherer, S., Ward, S., & Kim, S. K. (2000). Mol. Cell *6*, 605–616). Many of these ORF predictions have changed, and the current match is in column B. Column B, "wormbase gene name" gives the current gene prediction that is complementary to the probe on the array. Columns C-K are annotations downloaded from Wormbase in 2003, from April to June. The column headings are defined in Wormbase. Columns L and M are optimal DAF-16 binding sites, which are the sequence TTGTTTAC. Negative numbers are sites upstream of the start of translation, and positive numbers are downstream of the stop codon. These sites were predicted in Fall 2002 using data and software from  (van Helden, J., André, B., & Collado-Vides, J. (2000). Yeast *16*,177–187.). All sites within 2000 bp upstream and 300 bp downstream are shown, except for a few (<2%) that we were not able to match because of different annotation in our database and the rsat database. Columns N-O are our annotation of function based on sequence similarity, annotation from wormbase, and published reports. For the following groups, we have annotated all genes in the group, to the best of our knowledge: G protein coupled receptors, glutathione S transferases, cytochrome p450s, heat shock proteins, peroxidases, UGTs, epoxide hydrolases, collagens, cuticlins, NRF6 related, scl-1 familly (aka CRISP family), signaling proteins, and transcription factors. For the following groups, we have annotated only a subset of genes in the group: amine oxidases, ribosomal proteins, amino acid catabolism, and lipid metabolism. Column P, "mountains", lists groups of putatively co-regulated genes from Kim, S. K., Lund, J., Kiraly, M., Duke, K., Jiang, M., Stuart, J. M., Eizinger, A., Wylie, B. N., & Davidson, G. S. (2001). Science *293*, 2087–2092. Column Q and R list the average ratio of signal from *daf-c *genotypes to wild-type, using all experiments. Column Q is the average expressed as a base 2 logarithm (>0 means the expression was higher in *daf-c, *<0 is higher in N2, and Column R is the average expressed as a fold change (for both columns, a positive number means the expression was higher in *daf-c*, a negative number means the expression was higher in N2). Column S is the p value for the values in column Q and R, using a t test, asking the question, given the standard deviation for the data, is the value significantly different from a ratio of 1 (or 0 for the base 2 log). For these calculations we used the local standard deviation and the global standard deviation as described in Jiang, M., Ryu, J., Kiraly, M., Duke, K., Reinke, V., & Kim, S. K. (2001). Proc. Natl. Acad. Sci. *98, *218–223. Column T is the number of successful experiments for each spot on the array. The numbers vary because the data for a particular spot may or may not be of acceptable quality for a given experiment. Some genes have a number greater than 10 (the total number of independent experiments) because the same PCR product was put on the array in two different locations. Columns U thru BH are the data for each spot for each experiment. The column labeled "CH1D_MEAN" is the data for wild-type sample, the column labeled CH2D_MEAN is the data for the *daf-c *mutant sample, the column labeled CORR is the correlation coefficient for the average ratio of channel 1 to channel 2 calculated pixel by pixel, and the column labeled Flag indicates the data quality. Any value other than 0 indicates that the data had problems and was not used for analysis. Columns BL to CG give the data broken down by genotype (for each of the three daf-c genotypes used in this study). For each genotype, the first three or four columns give the base 2 log of the ratio of channel 2 (from *daf-c *genotype) to channel 1 (from wild type) for each experiment. Blank cells indicate bad data, not used in the analysis. The five digit number in the headings of these colums refer to the experiment ID used to catalog data at the Stanford Microarray Database. The next column gives the average ratio, the next column the standard deviation and the next gives the number of successful experiments, and the next the p value, asking the question, given the standard deviation for the data, is the value significantly different from a ratio of 1 (or 0 for the base 2 log). Click here for file

Additional File 2 Strongly regulated genes. Table S2 may be found online at:  Table S2 has data identical to table 1, except that only genes for which the regulation was greater than 2.145 fold, with a p value less than 0.01 are shown. Click here for file

Additional File 3 Regulated genes with putative DAF-16 binding sites. Table S3 can be found online at:  All data in this table are taken from Table S1. The first 15 rows have data for downregulated genes that have a daf-16 binding site downstream of the stop codon. The next 134 rows have data for genes that are upregulated and have a daf-16 binding site upstream of the start codon. Column A, "SMD gene name" is the name given to the probe on the microarray in the Stanford Microarray Database. The probe is a PCR product from a pair of primers made to amplify the ORF named in this column (Reinke, V., Smith, H. E., Nance, J., Wang, J., Van Doren, C., Begley, R., Jones, S. J. M., Davis, E., Scherer, S., Ward, S., & Kim, S. K. (2000). Mol. Cell *6*, 605–616). Many of these ORF predictions have changed, and the current match is in column B. Column B, "wormbase gene name" gives the current gene prediction that is complementary to the probe on the array. Columns C-G are annotations downloaded from Wormbase in 2003, from April to June. The column headings are defined in Wormbase. Columns H-J are optimal DAF-16 binding sites, which are the sequence TTGTTTAC. Negative numbers are sites upstream of the start of translation, and positive numbers are downstream of the stop codon. These sites were predicted in Fall 2002 using data and software from  (van Helden, J., André, B., & Collado-Vides, J. (2000). Yeast *16*,177–187.). All sites within 2000 bp upstream and 300 bp downstream are shown, except for a few (<2%) that we were not able to match because of different annotation in our database and the rsat database. Column K is our annotation of function based on sequence similarity, annotation from wormbase, and published reports. Our annotation of the following gene types is complete, to the best of our knowledge: G protein coupled receptors, glutathione S transferases, cytochrome p450s, heat shock proteins, peroxidases, UGTs, epoxide hydrolases, collagens, cuticlins, NRF6 related, scl-1 familly (aka CRISP family), signaling proteins, and transcription factors. The following groups are incompletely annotated: amine oxidases, ribosomal proteins, amino acid catabolism, and lipid metabolism. Column L, "mountains", lists groups of putatively co-regulated genes from Kim, S. K., Lund, J., Kiraly, M., Duke, K., Jiang, M., Stuart, J. M., Eizinger, A., Wylie, B. N., & Davidson, G. S. (2001). Science *293*, 2087–2092. Column M lists the average ratio of signal from *daf-c *genotypes to wild-type, using all experiments. expressed as a fold change (a positive number means the expression was higher in *daf-c*, a negative number means the expression was higher in N2). Column N is the p value for the values in column M, using a t test, asking the question, given the standard deviation for the data, is the value significantly different from a ratio of 1 (or 0 for the base 2 log). For these calculations we used the local standard deviation and the global standard deviation as described in Jiang, M., Ryu, J., Kiraly, M., Duke, K., Reinke, V., & Kim, S. K. (2001). Proc. Natl. Acad. Sci. *98*, 218–223. Click here for file

Additional File 4 Regulated regulatory genes. This table lists all transcription factors, signaling molecules, possible hormone biosynthetic enzymes, and other regulatory genes that are significantly regulated in our data. Click here for file
